# Non-health care costs associated with neonatal intensive care unit visitation

**DOI:** 10.1093/haschl/qxaf043

**Published:** 2025-02-28

**Authors:** Rebecca A Gourevitch, Evan Ellicott, Christine Kim, Maranna Yoder, Molly Passarella, Scott A Lorch, Michel Boudreaux

**Affiliations:** Department of Health Policy and Management, University of Maryland, College Park, MD 20742, United States; Department of Geographical Sciences, University of Maryland, College Park, MD 20742, United States; Department of Health Policy and Management, University of Maryland, College Park, MD 20742, United States; Department of Economics, University of Maryland, College Park, MD 20724, United States; Department of Pediatrics, Children's Hospital of Pennsylvania, Philadelphia, PA 19104, United States; Department of Pediatrics, Children's Hospital of Pennsylvania, Philadelphia, PA 19104, United States; Department of Health Policy and Management, University of Maryland, College Park, MD 20742, United States

**Keywords:** NICU care, children’s health, costs of care, costs of caregiving

## Introduction

Nearly 1 in 10 babies born in the United States is admitted to a Neonatal Intensive Care Unit (NICU). NICU care can be essential for improving outcomes and saving lives.^1^ The regionalization of NICU care has improved outcomes for high-risk neonates, but it requires many families to travel to visit their baby.^2^ This travel carries many ancillary costs, including transportation, lodging, childcare for other children, and unpaid leave from work.^3^ Studies have shown that these costs pose meaningful barriers to NICU visitation, an important practice that is associated with improved outcomes for both neonates and their mothers.^4,5^ However, no prior studies have assessed the magnitude and variation of these costs nationwide. In this study, we quantified the magnitude and variation of major non-health care costs associated with NICU visitation.

## Methods

We obtained the exact location of each level 2, 3, or 4 NICU from neonatologysolutions.com and used geospatial analysis to identify the closest NICU to the population-weighted centroid of every county in the continental US. We measured the driving distance from the centroid to the NICU, the associated drive time, and the mileage cost by multiplying the driving distance by the Internal Revenue Service standard mileage rate. We identified the cost of 8-hours of parking for patients at each NICU from hospital websites, or, if not available, by calling hospitals.

We used data from the Bureau of Labor Statistics (BLS) to identify the county's median hourly wage for all occupations and multiplied it by 8 h to reflect a day's forgone wages. We used BLS data on childcare worker hourly wages, multiplied by 8-hours, to estimate daily county-level childcare costs for an older child. We used the General Services Administration's county-specific reimbursement rates to estimate lodging and meal costs in the county of the closest NICU.

For each county, we aggregated these costs into daily estimates and scaled the total and component costs by the average NICU length of stay, 14 days.^6^ We used Rural-Urban Continuum Codes to classify counties as metropolitan, non-metropolitan but adjacent to a metropolitan area, or non-metropolitan and not adjacent to a metropolitan area. As most NICUs are in metropolitan areas, we considered metropolitan counties to be “close” to a NICU (see online Appendix Table S1 for alternative definitions). (To access the Appendix, click on the details tab in the article online). As people who live close to the NICU may not require overnight lodging, the total daily cost for metropolitan counties did not include lodging. Likewise, families that live far from the NICU may not travel round-trip daily; for non-metropolitan counties, the total 14-day cost includes only one round-trip drive.

We report descriptive statistics across counties and population-weighted means using each county's population of females of reproductive age from the 2023 American Community Survey, both overall and stratified by county rurality.

## Results

County centroids are an average of 90 miles from the closest NICU, or a 102-min drive with $61 of associated mileage expenses (Table 1). Each day of NICU visitation has an average cost of $209 in forgone wages, $117 in lodging, and $62 in meals. The daily average cost of childcare is $110. Parking was free at most NICUs. As most women of reproductive age live in metropolitan areas, the population-weighted driving time and associated expenses are lower, but other costs are higher.

**Table 1.** Travel time and common expenses incurred for NICU visitation, daily and for average-length of stay (weighted and unweighted).

	Unweighted costs				Weighted costs	
	Daily mean	Daily median	Daily IQR	14-Day mean	Daily mean	14-Day mean
Drive distance (miles)	90	73	(39, 122)	1265	25	348
Drive time (minutes)	102	87	(51, 134)	1426	36	497
Forgone wages	$209	$201	($188, $221)	$2923	$248	$3466
Lodging cost	$117	$107	($107, $116)	$1641	$141	$1979
Childcare cost	$110	$108	($99, $116)	$1540	$125	$1745
Drive cost	$61	$49	($26, $82)	$848	$17	$233
Meals cost	$62	$59	($59, $64)	$863	$66	$929
Parking cost	$0	$0	($0, $0)	$7	$3	$41
Full day cost	$513	$510	($442, $563)	$6510	$472	$6511

Data is shown at county-level for 3108 counties or county equivalents in the continental US. We assumed that a family travels to the closest NICU to the population-weighted centroid of their county. Mileage cost is 2024 Internal Revenue Service standard mileage rate. Parking costs were collected by contacting each hospital in March-May 2024 (data were obtained for 800 of the 814 NICUs). Data on wages and childcare costs are from the BLS (May 2023). Data on lodging, mileage, and meals cost are General Services Administration standard reimbursement rates (October 2023-September 2024). Estimates are scaled for round-trip driving and 8-hours of visiting per day. Lodging and meals costs are for the nearest NICU's county. Weighted costs use the county's population of females of reproductive age (15-44) from the 2023 American Community Survey.

This amounts to an average daily cost of $513 ($472, weighted). For an average-length NICU stay of 14-days,^6^ the average cost would be $6510 ($6511, weighted)—or approximately 10% of annual median household income.

There was wide variation in costs across counties (online Appendix Figure S1); 25% were at or below a $442 average daily cost, and 25% were at or above $563. The average daily cost was $586 in non-metropolitan, non-metropolitan adjacent areas and $433 in metropolitan areas, corresponding to farther drive distances (online Appendix Table S2).

## Discussion

We present the first nationwide estimates of ancillary costs of NICU visitation, finding that families in an average county face $6466 over an average-length NICU stay, with those farther from NICUs facing higher costs due to travel distance. For many of the over 300 000 families with NICU stays annually, this represents an unaffordable cost that may limit their engagement in care or require disruption to household finances during an otherwise vulnerable period.

These potential consequences require policy solutions. While some hospitals and private organizations have programs to offset these ancillary costs, there are no systematic policies to make NICU visitation more affordable. We found that forgone wages were the largest cost driver, suggesting that paid family leave would substantially offset these costs. Policies that provide cash assistance, especially to low-income families, are an evidence-based option to improve visitation and allow families to use the support where it is most needed.^7^

Our findings highlight that long travel distances increase costs. Importantly, we describe travel to the closest level 2, 3, or 4 NICU. However, regionalization requires families of infants needing higher levels of care to travel farther and face longer lengths of stay. This carries more associated expenses, and therefore these families may need additional supports.

Our study is limited by using county-level data. Not all included costs apply to all families. For example, some families may rely on public transportation to travel to the NICU. Future work should quantify families' realized costs of NICU visitation and document the impact of efforts to offset these costs.

## Supplementary Material

qxaf043_Supplementary_Data
