# Supplementary material for: Non-health care costs associated with neonatal intensive care unit visitation
Source: Health Aff Sch. 2025 Feb 28;3(3):qxaf043. doi: 10.1093/haschl/qxaf043 (PMC11909500; doi:10.1093/haschl/qxaf043)
Supplement: qxaf043_Supplementary_Data [file qxaf043_supplementary_data.zip › NICU_Cost_Letter_HAScholar_appendix.docx]

**Appendix**

**Appendix Table 1**. Full Day Cost Calculations for Alternative Definitions of Counties Considered Driving Distance from the Nearest NICU

| *Counties considered driving distance from nearest NICU* | **Unweighted Total Costs** | | | | **Weighted Total Costs** | |
| --- | --- | --- | --- | --- | --- | --- |
|  | Daily  Mean | Daily Median | Daily  IQR | 14-Day Mean | Daily Mean | 14-Day Mean |
| Metropolitan counties | $513 | $510 | ($442, $563) | $6,510 | $472 | $6,511 |
| Counties with < 30 miles between centroid and nearest NICU | $511 | $518 | ($422, $573) | $6,466 | $473 | $6,506 |
| Counties with < 60 miles between centroid and nearest NICU | $472 | $431 | ($398, $542) | $6,170 | $462 | $6,419 |
| Counties with < 120 miles between centroid and nearest NICU | $448 | $428 | ($398, $470) | $6,127 | $459 | $6,410 |

*Notes*: Metropolitan counties were those with 2023 Rural-Urban Continuum Codes (RUCC) indicating a metropolitan area (RUCC 1, 2, or 3).

**Appendix Figure.** NICU Locations and County Variation in Total Approximate Daily Cost of NICU Visitation


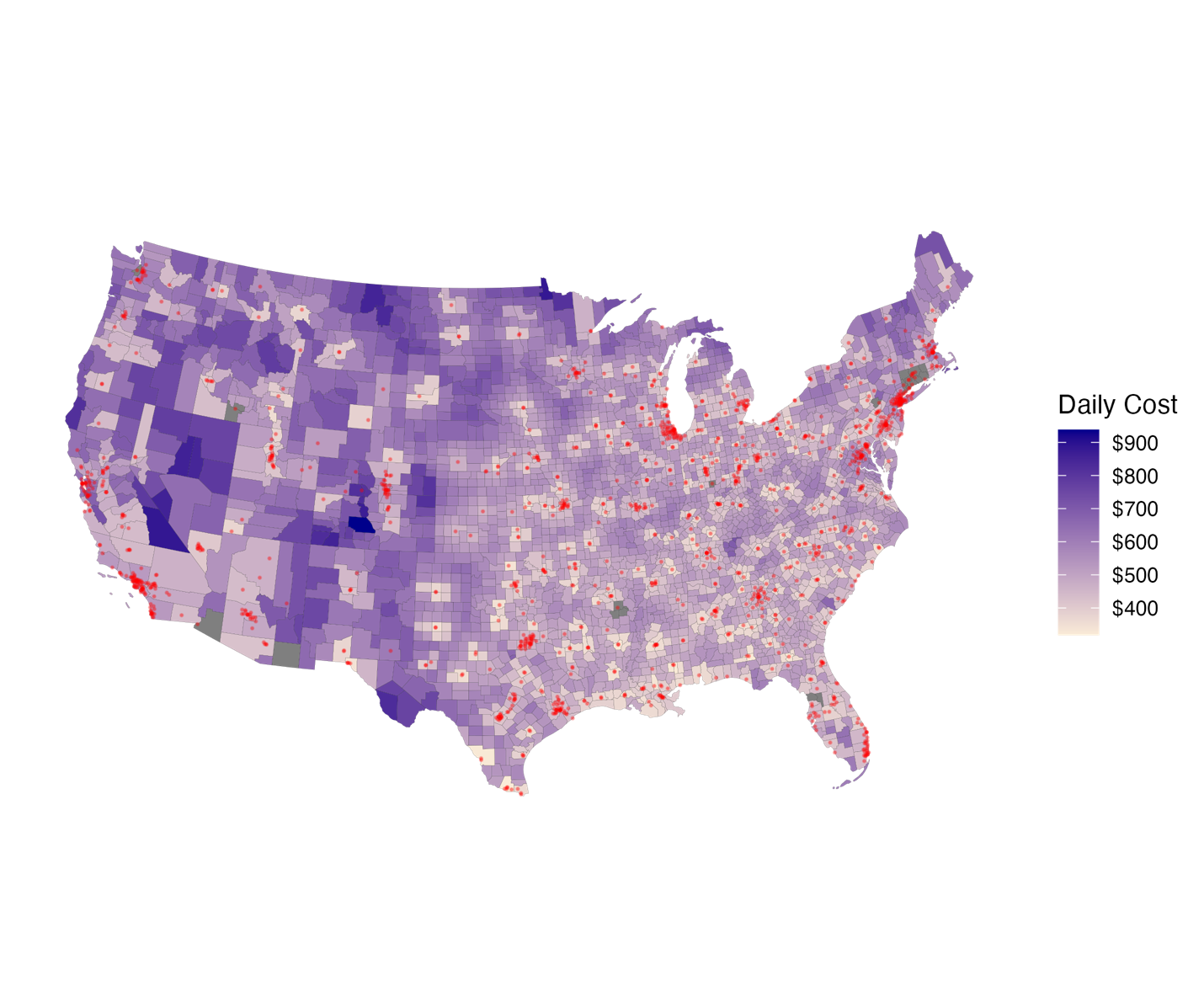


*Notes*: Red dots indicate NICU locations. Cost data are the full cost estimates as reported in the main text exhibit. Grey indicates that childcare cost estimates were not available for that county (N=12 counties).

**Appendix Table 2.** Travel Time and Common Expenses Incurred for NICU Visitation, Daily and for Average Length of Stay (Weighted and Unweighted), by County Rural-Urban Continuum Code Group

|  | **Unweighted Costs** | | | | **Weighted Costs** | |
| --- | --- | --- | --- | --- | --- | --- |
|  | Daily  Mean | Daily Median | Daily  IQR | 14-Day Mean | Daily Mean | 14-Day Mean |
| **Metropolitan** **Counties** (N=1,180) | | | | | | |
| Drive Distance (Miles) | 37 | 31 | (10, 56) | 517 | 15 | 209 |
| Drive Time (Minutes) | 49 | 44 | (20, 70) | 688 | 25 | 353 |
| Forgone Wages | $230 | $222 | ($204, $244) | $3,214 | $255 | $3,565 |
| Lodging Cost | $122 | $107 | ($107, $126) | $1,709 | $145 | $2,030 |
| Childcare Cost | $114 | $112 | ($103, $122) | $1,602 | $127 | $1,777 |
| Drive Cost | $25 | $21 | ($6, $38) | $347 | $10 | $140 |
| Meals Cost | $63 | $59 | ($59, $64) | $882 | $67 | $939 |
| Parking Cost | $1 | $0 | ($0, $0) | $12 | $3 | $46 |
| Full Day Cost | $433 | $425 | ($394, $460) | $6,058 | $462 | $6,468 |
| **Non-Metropolitan, Metropolitan-Adjacent Counties** (N=1,044) | | | | | | |
| Drive Distance (Miles) | 91 | 85 | (63, 113) | 1,274 | 79 | 1,108 |
| Drive Time (Minutes) | 104 | 99 | (76, 125) | 1,457 | 93 | 1,301 |
| Forgone Wages | $196 | $195 | ($184, $204) | $2,746 | $197 | $2,757 |
| Lodging Cost | $114 | $107 | ($107, $111) | $1,602 | $114 | $1,602 |
| Childcare Cost | $107 | $105 | ($96, $114) | $1,496 | $108 | $1,518 |
| Drive Cost | $61 | $57 | ($42, $75) | $854 | $53 | $742 |
| Meals Cost | $61 | $59 | ($59, $64) | $856 | $61 | $860 |
| Parking Cost | $0 | $0 | ($0, $0) | $5 | $1 | $8 |
| Full Day Cost | $540 | $525 | ($502, $564) | $7,561 | $535 | $7,493 |
| **Non-Metropolitan, Non-Metropolitan-Adjacent Counties** (N=884) | | | | | | |
| Drive Distance (Miles) | 161 | 149 | (101, 205) | 2,254 | 131 | 1,838 |
| Drive Time (Minutes) | 170 | 158 | (114, 215) | 2,376 | 144 | 2,014 |
| Forgone Wages | $196 | $195 | ($186, $206) | $2,743 | $197 | $2,757 |
| Lodging Cost | $114 | $107 | ($107, $107) | $1,597 | $117 | $1,633 |
| Childcare Cost | $108 | $105 | ($99, $115) | $1,509 | $109 | $1,526 |
| Drive Cost | $108 | $100 | ($68, $137) | $1,510 | $88 | $1,232 |
| Meals Cost | $61 | $59 | ($59, $59) | $848 | $61 | $852 |
| Parking Cost | $0 | $0 | ($0, $0) | $2 | $0 | $2 |
| Full Day Cost | $586 | $571 | ($527, $632) | $8210 | $572 | $8,002 |

*Notes*: Metropolitan counties were those with 2023 Rural-Urban Continuum Codes (RUCC) indicating a metropolitan area (RUCC 1, 2, or 3). Non-Metropolitan, Metropolitan-Adjacent counties correspond to codes 4, 6, or 8, and Non-Metropolitan, Non-Metropolitan-Adjacent areas had RUCC codes 5, 7, or 9.
